# Retrieval practice facilitates learning by strengthening processing in both the anterior and posterior hippocampus

**DOI:** 10.1002/brb3.1909

**Published:** 2020-10-22

**Authors:** Carola Wiklund‐Hörnqvist, Sara Stillesjö, Micael Andersson, Bert Jonsson, Lars Nyberg

**Affiliations:** ^1^ Department of Psychology Umeå University Umea Sweden; ^2^ Umeå Center for Functional Brain Imaging (UFBI) Umeå University Umea Sweden; ^3^ Department of Applied Educational Science Umeå University Umea Sweden; ^4^ Department of Integrative Medical Biology Umeå University Umea Sweden; ^5^ Department of Radiation Sciences Umeå University Umea Sweden

**Keywords:** hippocampus, learning and memory, retrieval practice, the testing effect

## Abstract

**Introduction and Methods:**

A large number of behavioral studies show that retrieval practice is a powerful way of strengthening learning of new information. Repeated retrieval might support long‐term retention in a quantitative sense by inducing stronger episodic representations or in a qualitative sense by contributing to the formation of more gist‐like representations. Here we used fMRI to examine the brain bases related to the learning effects following retrieval practice and provide imaging support for both views by showing increased activation of anterior and posterior hippocampus regions during a delayed memory test.

**Results:**

Brain activity in the posterior hippocampus increased linearly as a function of number of successful retrievals during initial learning, whereas anterior hippocampus activity was restricted to items retrieved many but not few times during the learning phase.

**Conclusion:**

Taken together, these findings indicate that retrieval practice strengthens subsequent retention via “dual action” in the anterior and posterior hippocampus, possibly reflecting coding of individual experiences as well as integration and generalization across multiple experiences. Our findings are of educational significance by providing insight into the brain bases of a learning method of applied relevance.

## Graphical abstract

The hippocampus (HC) and the “testing effect” have each been studied extensively for their potential in memory and learning. Here we provide novel evidence related to the well‐established “testing effect” by highlighting that retrieval practice facilitates learning by strengthening processing in both the anterior and posterior HC. These findings are of educational significance by providing unique insights into the brain bases of a learning method of applied relevance and emphasize the important role of hippocampus for durable learning in school.

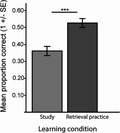

## 1 INTRODUCTION

Repeatedly testing yourself while learning new information (i.e., *retrieval practice*) improves retention of the to‐be‐learned material more than typical repetition or *restudy* (Roediger & Butler, 2011; Roediger & Karpicke, 2006a, 2006b; Rowland, 2014). This boost of learning from retrieval practice is commonly referred to as the *testing effect* (TE; Karpicke & Roediger, 2008; Roediger & Butler, 2011; Roediger & Karpicke, 2006a, 2006b; Rowland, 2014). Cognitive accounts (Karpicke et al., 2014; Lehman et al., 2014; Rickard & Pan, 2017) and behavioral studies of the TE (e.g., Jang et al., 2012; Rawson & Dunlosky, 2011; Roediger & Butler, 2011; Rowland, 2014; Vaughn & Rawson, 2011) have attributed the benefits of retrieval practice to memory strength, emphasizing that a critical aspect relates to the number of successful repeated retrievals during learning. For example, Vaughn and Rawson (2011) found that students who practiced retrieval until the items were recalled four to five times versus only once performed significantly better at the final test measured one week later (see also Rawson & Dunlosky, 2011 for related findings). Such findings align well with cognitive explanations suggesting that successful retrievals during retrieval practice strengthen the association between the stimuli and the response via a “test memory” (Rickard & Pan, 2017), and this ongoing process restricts the search set and increases the likelihood of successfully recovering a target in the future (Karpicke et al., 2014; Lehman et al., 2014; Racsmány et al., 2018). While behavioral evidence and cognitive explanations have been proposed for the TE, considerably less is known about its brain basis (van den Broek et al., 2016). The hippocampus (HC) region is a gateway to new learning (Eichenbaum, 2017). A few studies of the TE found HC activity during retrieval practice to be predictive of subsequent memory when contrasted with study (Jonker et al., 2018; Liu et al., 2014; Wing et al., 2013), and higher HC activity was seen for items successfully remembered one week after retrieval practice (Karlsson Wirebring et al., 2015). However, other studies examining the TE failed to observe differential HC recruitment (Keresztes et al., 2013; van den Broek et al., 2013). Thus, conclusive evidence for a role of the HC in the TE is lacking.

To deepen our understanding of the role of the HC in the TE, it might be informative to consider the emerging notion of a functional differentiation along the long axis of the HC (Dandolo & Schwabe, 2018; Poppenk et al., 2013; Strange et al., 2014). Functional imaging studies have shown that both aHC and pHC are involved in encoding and retrieval, with greater anterior (aHC) activity at encoding and greater posterior (pHC) activity at retrieval (Grady, 2019; Kim, 2015; Nyberg et al., 2019). Differences along the hippocampal longitudinal axis have also been linked to the nature of mnemonic representations. Several studies link aHC to more abstract representations across multiple experiences (e.g., Bowman & Zeithamova, 2018; Brunec et al., 2018; Frank et al., 2019) and pHC to the coding of individual experiences (Collin et al., 2015; Poppenk et al., 2013; for partly conflicting findings, see Dandolo & Schwabe, 2018).

**FIGURE 1:**
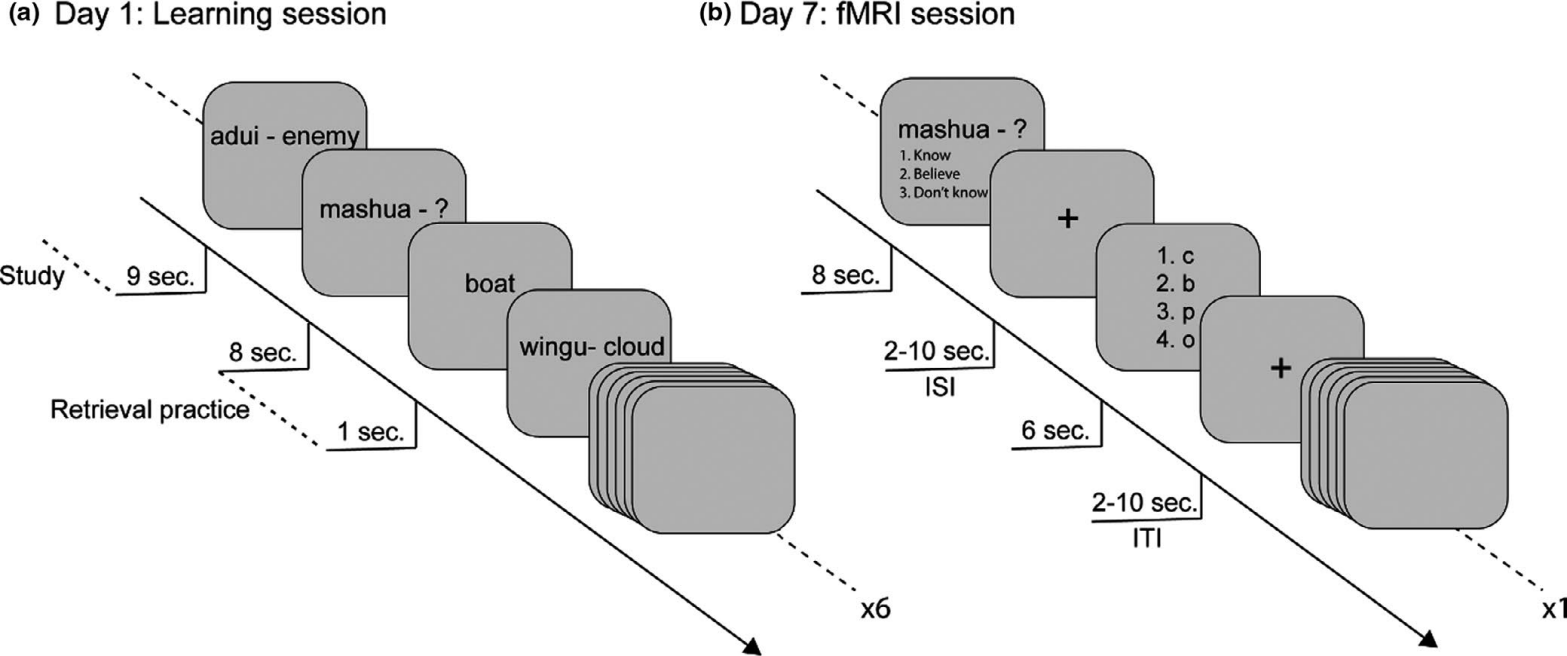
(a–b) Displays the trial procedure at day 1 (a) and one week after learning (b). In panel a, the gray squares represent the presentation order of the randomly interspersed word‐pairs for the study and retrieval practice with feedback condition. (a) At day 1, for retrieval practice items, the Swahili word appeared on the screen with a question mark [mashua—?] and the participants were asked to type in the corresponding Swedish counterpart at their computer (max 8 s), followed by immediate feedback (1 s; [boat]). For study items, the intact Swahili‐Swedish word‐pair was presented on the screen (9 s [adui—enemy]). In panel b, the squares represent the trial procedure of the cued‐recall test performed in the scanner (see Material and Procedure section for details)

Here we used event‐related functional magnetic resonance imaging (fMRI) to examine the brain bases of the TE, with special focus on the HC. We included a sample of upper secondary‐school students (*N* = 50). Although the testing effect has been demonstrated even in preschool children (Fritz et al., 2007), the majority of previous studies from our group (e.g., Wiklund‐Hörnqvist et al., 2017) and others (Schwieren et al., 2017) involved introductory university students. The to‐be‐learned material was Swahili‐Swedish word‐pairs (Karlsson Wirebring et al., 2015; Wiklund‐Hörnqvist et al., 2017). In the classroom, students learned half of the word‐pairs by study and the other half by retrieval practice with feedback. In both conditions, each word‐pair was repeatedly presented six times (see Figure 1a). One week after the learning session, the students were given a cued‐recall test of the previously learned word‐pairs in the MR scanner (see Figure 1b and detailed methods section, see also Karlsson Wirebring et al., 2015 for a similar design).

First, we predicted higher activity in the HC for remembered words learned through retrieval practice relative those learned through study. Second, motivated by behavioral studies emphasizing that a factor underlying the testing effect is the number of successful repeated retrievals during learning (e.g., Jang et al., 2012; Rawson & Dunlosky, 2011; Roediger & Butler, 2011; Rowland, 2014), we predicted that HC activity would scale with the number of successful retrievals during learning at day one (i.e., the initial learning session). For both analyses, potential differences along the HC axis were assessed. If aHC activity reflects more abstract representations across multiple experiences (e.g., Bowman & Zeithamova, 2018; Brunec et al., 2018; Frank et al., 2019), then we expect that activity in aHC should be more pronounced for items that were retrieved many (>three times, Vaughn & Rawson, 2011) versus few times (≤three times) during the initial learning session. If pHC activity reflects detailed representations that are strengthened by testing during the initial phase, then pHC activity might be a general signature of the TE that, in contrast to aHC, is more gradually strengthened by the number of successful retrievals during the learning session.

## 2 METHODS

### 2.1 Participants

Fifty neurologically healthy students from the third year in upper secondary school (*M*_age_ = 17.9 years; age range 17–19, 25 males) participated in the study. All participants were native Swedish speakers, right handed by self‐report, had normal or corrected‐to‐normal vision, and no participant reported prior experience with the Swahili language. For subjects (*n* = 8) who had not yet attained a legal age of majority (18 years), written informed consent was obtained from the participant and both parents. The study was conducted in accordance with the Helsinki declaration and approved by the Regional Ethics Committee at Umeå University, Sweden.

### 2.2 Material and procedure

The to‐be‐learned material consisted of 60 Swahili‐Swedish word‐pairs translated from Nelson and Dunlosky (1994) and previously used (e.g., Karlsson Wirebring et al., 2015; Wiklund‐Hörnqvist et al., 2017). One week prior the scanning session, a computerized within‐subject learning session of 60 Swahili‐Swedish word‐pairs was conducted in the classroom (see Figure 1a). First, to familiarize the participants with the to‐be‐learned material, all Swahili‐Swedish word‐pairs were presented one by one on the computer screen once, before the learning phase started. Next, across six consecutive runs, half of the word‐pairs were learned though retrieval practice (cued recall; [mashua—?]) followed by feedback (correct answer; [boat]), and the other half through study [mashua‐boat] (see Figure 1a). To prevent item and order effects, retrieval practice items and study items were randomly interspersed during the learning session, and each participant had a unique learning list.

One week after the learning session, participants were invited to perform the one week cued‐recall test of all 60 Swahili‐Swedish word‐pairs in the MR scanner (see Figure 1b). In order to examine the TE, all 60 Swahili‐Swedish word‐pairs were tested once in the MR scanner and the trial procedure used was partly the same as in prior studies (day 7, Karlsson Wirebring et al., 2015; Wiklund‐Hörnqvist et al., 2017). As can be seen in Figure 1b, participants received the Swahili word as a cue and were asked to recall the Swedish counterpart. The Swahili word was shown for a maximum of 8 s. Within this time, participants were asked to respond by pressing a four button keypad with their right hand fingers to indicate whether they had recalled a Swedish word they (a) “Knew was correct” (index finger), (b) “Believed was correct” (middle finger), or (c) “Did not retrieve a word” (ring finger). Next, a jittered crosshair (ISI, 2–10 s.) appeared on the screen. Participants were then asked to choose among four alternatives for the second letter in the Swedish word they had just retrieved, using the right hand fingers, within 6 s. The second letter was used to single out the correctly remembered words labeled as successfully remembered. The position of the correct answer relative to the lures was systematically varied, such that the target appeared equally often in each of the four possible positions (see Karlsson Wirebring et al., 2015; Wiklund‐Hörnqvist et al., 2017 for a related experimental setup). This was followed by a jittered crosshair (ITI, 2–10 s) before the next probe appeared on the screen (see Figure 1b). The session ended with structural imaging. In total, the scanning session lasted for ~40 min. Immediately after the scanning session, all participants completed a postscan confirmatory test (a list with all 60 Swahili words) by paper and pencil outside the scanner. In the confirmatory test, all participants were asked to fill in the Swedish word of those they classified as “know/believe is correct” in the scanner.

### 2.3 Image acquisition and preprocessing

All scanner parameters used for image acquisition were identical as in a previous study focusing on retrieval practice (Karlsson Wirebring et al., 2015). Functional data were preprocessed and analyzed in SPM12 (www.fil.ion.ucl.ac.uk/spm; The Wellcome Centre for Human Neuroimaging, London, UK) assisted by an in‐house program (DataZ), run on MATLAB R2014b (MathWorks Inc, Natick, MA, USA). Images were corrected for slice timing and movement corrected with realign & unwarp. The T1‐images were segmented and a group template and individual flowfield files were created with DARTEL (Ashburner, 2007), which were used to normalize images to MNI space (2 mm), and then smoothed (8 mm FWHM Gaussian filter kernel). Statistical analyses were conducted on the smoothed data with a high‐pass filter (128 s. cutoff period) to remove low‐frequency noise.

### 2.4 Behavioral data and statistical analysis

Items responded as “know” or “believed” accompanied by an accurate response of the second letter in the Swedish word were scored as words successfully remembered, and analyzed in relation to how they were learned at day 1 (study/retrieval practice). Responses scored as successfully retrieved in the scanner were highly correlated with the postscan confirmatory test performance for the same item (*r* = .91, *p* < .001).

### 2.5 Imaging data and statistical analysis

Two fMRI analyses were of interest. First, we wanted to examine the TE (remembered after retrieval practice > remembered after study) in a whole‐brain analysis. Second, we wanted to identify brain regions sensitive to the number of successful retrievals during learning at day one by the use of a whole‐brain parametric analysis. As the main interest was on hippocampal activity, the above whole‐brain exploratory analyses were followed up by ROI analyses with the purpose to directly compare aHC with pHC. The ROI analyses were motivated by recent research indicating a functional differentiation along the hippocampal long axis (e.g., Brunec et al., 2018; Dandolo & Schwabe, 2018; Poppenk et al., 2013).

Four linear models for fMRI analyses were set up. First, one critical fMRI contrast concerned items successfully retrieved on day 7 in relation to prior learning on day 1 (study versus retrieval practice). The statistical model had regressors for items successfully retrieved related to study and retrieval practice, respectively. Onsets were defined as the beginning of the presentation of the cue (i.e., the Swahili word), durations were set to zero (but control analyses were added which considered response times), and the regressor was convolved with the canonical HRF. Movement parameters were included as regressors of no interest. The BOLD signal was high‐pass filtered with a 128s cutoff prior to regression. A contrast image of retrieval success effect estimation related to prior learning activity (remembered retrieval practice > remembered study) was made. Second, we ran the data analysis both with the main‐model defined above and with an additional model including the two regressors of interest (retrieval success related to study and retrieval practice, respectively), six regressors of no interest (forgotten trials, second letter retrieval success, second letter forgotten items, related to study and retrieval practice, respectively) along with the six movement parameters. The main findings were virtually identical regardless of model (see Figure S1).

Third, a regressor defined as linear parametric changes in fMRI signal as a function of number of correct retrievals during day 1 (range 1–6) in the retrieval practice condition was included in an additional model. Again, the head movement parameters were included in the model as regressors of no interest, and the regressor of interest was convolved with a hemodynamic response function. A contrast image of the parametric modulation was made for each subject at the first level. A fourth model was set up where the cue presentation onset times were grouped according to the number of correct retrievals during day 1. One regressor of interest per group was set up and one for study plus the six movement parameters were added as nuisance regressors. All but the movement parameters were events and convolved with a hemodynamic response function.

For model one and four, hippocampal ROI analyses were performed by calculating median of beta values (slope values from the regression) over voxels within the ROIs. One ROI for aHC and one for pHC were achieved by masking out the hippocampus and using an anterior–posterior border at *y* = −21 (Poppenk et al., 2013). The hippocampus mask was achieved by a freesurfer segmentation (https://surfer.nmr.mgh.harvard.edu/; Fischl et al., 2002, 2004) of the mean image of MNI‐normalized structural images of all subjects in the study and collapsed across hemispheres. The beta values were loaded into SPSS 25 (IBM Corporation, Armonk, NY, USA). For model one, a 2 (Hippocampal ROI: anterior, posterior) × 2 (Learning condition: retrieval practice, study) ANOVA was set up. For model four, a 2 (Hippocampal ROI: anterior, posterior) × 6 (Number of successful retrievals at day one: 1, 2, 3, 4, 5, 6) ANOVA was set up. In the latter, missing values were imputed by mean values for the group.

The contrast images from model one and two were then used in two one‐sample *t* tests. For the group analysis related to the TE, we used a voxel‐level threshold at *p* < .001 uncorrected, with cluster‐level threshold *p* < .05 FWE‐corrected. For the parametric modulation approach, four subjects were excluded due to registration failures of data (i.e., data were not completely recorded after the completed learning session) at day 1, and we set a voxel‐level threshold at *p* < .01 FDR‐corrected, with a cluster‐level threshold *p* < .05 FWE‐corrected. For visualization of the effects by the whole‐brain parametric modulation analysis, mean BOLD signal related to the onset of the cue (day 7) is plotted in relation to the number of successful retrievals (day 1).

## 3 RESULTS

**FIGURE 2:**
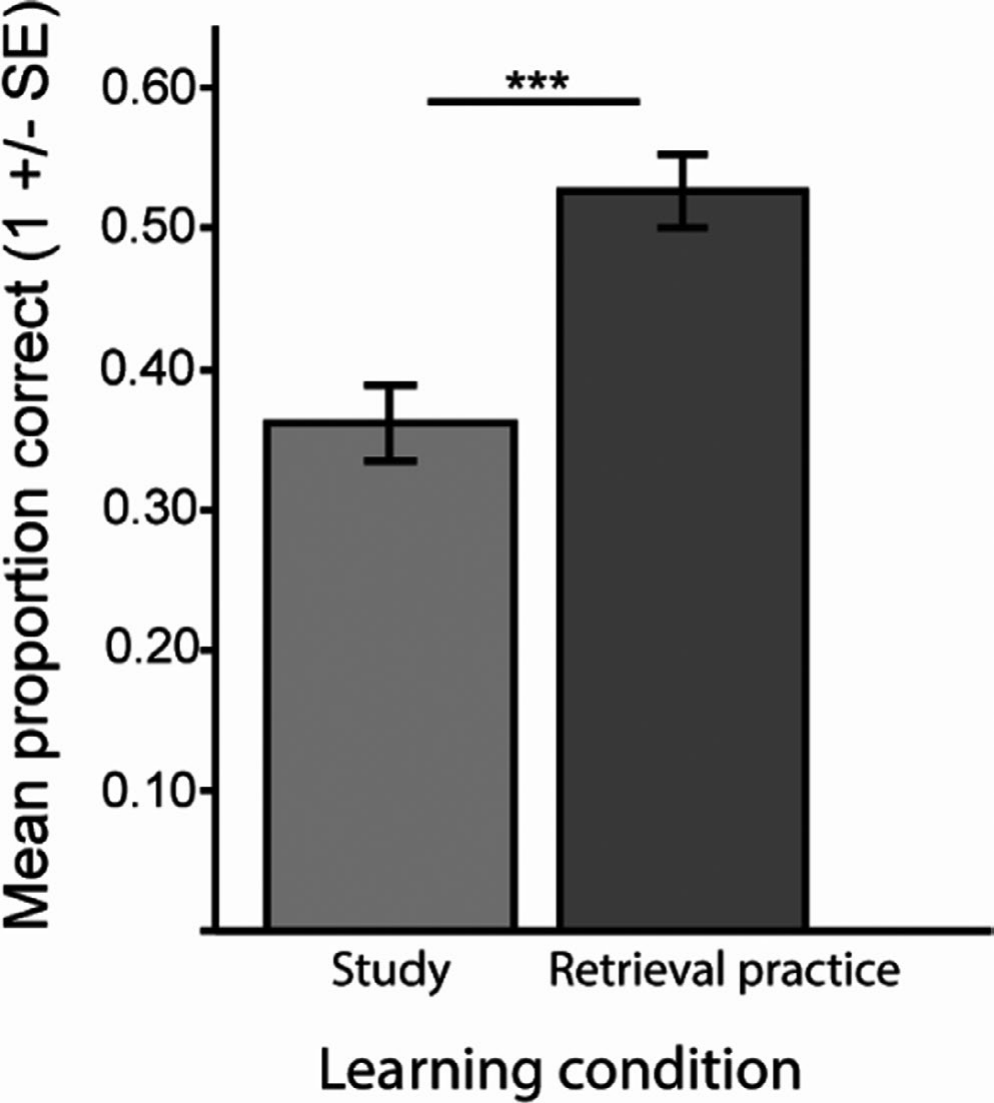
The behavioral TE. The mean proportion of correct responses in the MR scanner one week after the learning session. Error bars denote ± 1 *SEM*. ***p < .001

A paired *t* test confirmed a significant behavioral TE, *t* (49) = 8.61, *p* < .0001, Cohens *d* = 1.22, with the mean proportion correctly remembered items after retrieval practice = 0.53 (*SE* = 0.03) and after study = 0.36 (*SE* = 0.03) (see Figure 2).

**FIGURE 3:**
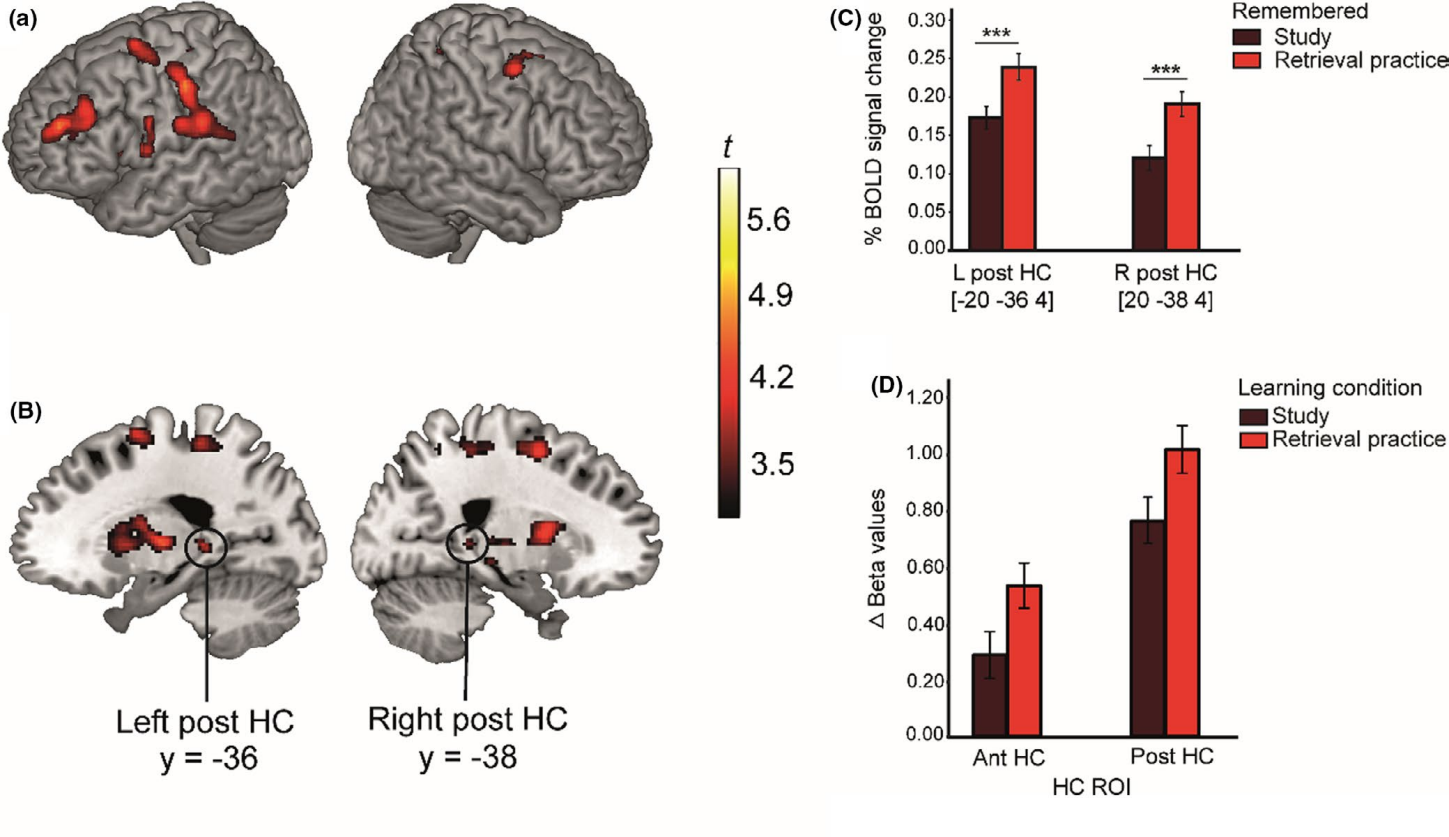
(a–d) Brain activation related to the whole‐brain TE contrast (remembered retrieval practice >remembered study) in the (a) whole brain, (b) in the bilateral posterior HC, (c) visualization of differences in the BOLD signal related to prior learning activity in the bilateral posterior HC, and (d) the results from the ROI analysis when comparing the average contribution in aHC with pHC collapsed across hemispheres related to prior learning condition. Error bars denote ±1 *SEM*. ***p < .001

After demonstrating a significant TE, we analyzed fMRI responses to items successfully remembered during the cued‐recall test as a function of how they were learned one week prior the scanning session. It should be stressed that this whole‐brain analysis contrasted “items correctly retrieved versus items correctly retrieved”—only the initial learning processes differed (retrieval practice vs. study). Higher BOLD signal after retrieval practice was observed in several regions, notably in the left hemisphere (Figure 3a, Table S1). In support of our prediction of a role of the HC in the TE, we observed higher activity in bilateral pHC for retrieval practice compared to study (see Figure 3b and c). The bilateral activation in pHC remained when controlling for individual differences in the magnitude of the TE (proportion correct retrieval practice—proportion correct study; see Figure S2a and b). The reversed contrast (study > retrieval practice) revealed no significant clusters at the predefined statistical threshold.

Next, to directly compare the aHC and pHC, we conducted a 2 (Hippocampal ROI: anterior, posterior) × 2 (Learning condition: retrieval practice, study) repeated measures ANOVA based on the beta values extracted from the predefined HC ROIs collapsed across hemispheres (see Methods section for details). Significant main effects were found for HC ROI [*F* (1,49) = 130.46, *p* < .0001, *n* ^2^_p_ = 0.73] and learning condition [*F* (1,49) = 7.54, *p* = .008, *n* ^2^_p_ = 0.13], but no significant interaction between HC ROI and learning condition (*p* = .90) was evident (see Figure 3d). Thus, while the whole‐brain analysis identified a TE response in the pHC but not in the aHC, the ROI analysis indicated similarities in response patterns.

**FIGURE 4:**
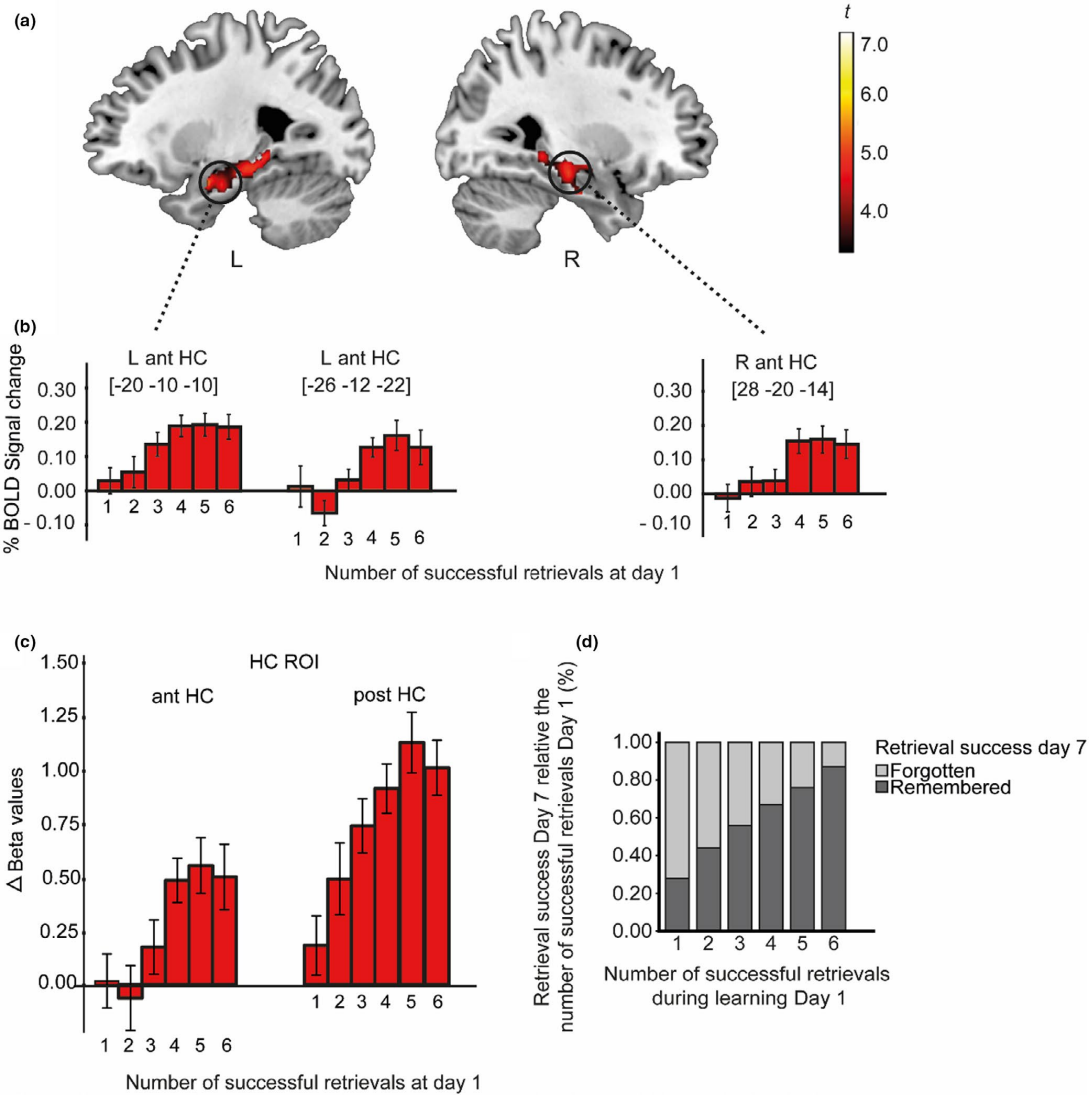
(a–d) Linear parametric modulation effects from the whole‐brain analysis in (a) left and right anterior HC as a function of the number of correct retrievals during day 1 (1, 2, 3, 4, 5, 6). (b) For visualization of the effects by the parametric modulation analysis, mean BOLD signal related to the onset of the cue (day 7) is plotted in relation to the number of successful retrievals during initial learning (day 1) in left and right anterior HC, respectively (see Figure S4 for bilateral pHC). (c) Linear parametric modulation effects in the predefined ROIs for aHC and pHC collapsed across hemispheres. Error bars denote ±1 *SEM*. (d) The mean proportion of remembered/forgotten items at day 7, relative the number of successful retrievals at day 1

Next, we implemented a whole‐brain parametric analysis to identify brain regions sensitive to the number of repeated successful retrievals during initial learning (i.e., day one). The results showed that activity in several peaks within the bilateral aHC was modulated as a function of number of successful repeated retrievals (see Figure 4a and b; see also Figure S3 and Table S1). The parametric modulation analysis also identified the left [−16 –32 –12; −28 –30 –12; −22 –38 –6] and right [20 2 –22 & 22 –16 –26] parahippocampal cortex.

Using the predefined hippocampal ROIs, a 2 (Hippocampal ROI: anterior, posterior) × 6 (Number of successful retrievals at day one: 1–6) repeated measures ANOVA was performed. Significant main effects of HC ROI [*F* (1,45) = 90.73, *p* < .0001, *n* ^2^_p_ = 0.67] and the number of successful retrievals [*F* (5, 225) = 7.12, *p* < .001, *n* ^2^_p_ = 0.14] were seen, along with a significant interaction between HC ROI and number of successful retrievals [*F* (5,225) = 3.96, *p* = .002, *n* ^2^_p_ = 0.08] (see Figure 4c; see also Figure 4d for related behavioral responses).

Taken together, as can be seen in Figure 4c, the pHC activity was apparent after only 1 successful retrieval during initial learning and then scaled linearly as a function of additional retrievals, whereas aHC activity adhered to a “threshold pattern” with stable activation after 4–6 retrievals (Figure 4b and c).

## 4 DISCUSSION

Our findings provide novel information on the brain bases of the effectiveness of retrieval practice. Correctly remembering information that one week earlier had been acquired by means of retrieval practice was associated with elevated BOLD signal in several left hemisphere regions that have been linked to language processing and semantic retrieval (Figure 3a; Binder & Desai, 2011; Cabeza & Nyberg, 2002; Martin & Chao, 2001; Price, 2012). This finding might be interpreted to show that initial retrieval practice transformed subsequent memory retrieval of Swahili‐Swedish word‐pairs to be more semantic than episodic in nature, possibly via rapid consolidation (Antony et al., 2017), although this interpretation must be verified in future research. In line with more recent cognitive explanations for the TE, the observed left hemisphere differences in regional BOLD signal could reflect differences in memory strength following different learning methods (Rickard & Pan, 2017; Roediger & Butler, 2011). Reversing the TE contrast yielded no significant effects.

Our main focus in the study was on the role of the HC in the TE. The initial whole‐brain analysis identified a strong response in the bilateral pHC when successful retrieval following initial retrieval practice was contrasted with successful retrieval following study only. The supplementary ROI‐based analysis confirmed this pHC effect and also revealed a similar, albeit weaker, TE response in the aHC. Thus, these findings support past observations that retrieval practice strengthens HC involvement (Karlsson Wirebring et al., 2015). The parametric whole‐brain and ROI analyses provided further insight into the role of the HC in the TE by indicating that the aHC involvement was dependent on items having been retrieved multiple times during the initial learning session, whereas pHC involvement was seen even for items only retrieved once during the initial session but then gradually increased as a function of successful retrievals. This response pattern is in line with the view that the aHC supports formation of more abstract representations that emerge after multiple experiences (e.g., Bowman & Zeithamova, 2018; Brunec et al., 2018; Frank et al., 2019), and that the pHC is involved in the coding of individual experiences (Collin et al., 2015; Poppenk et al., 2013).

This hypothetical “dual action” of both aHC and pHC contributions, speculatively contributing both more gist‐like mnemonic as well as more detailed representations after retrieval practice, could explain the very robust effectiveness of retrieval practice across study materials and populations (Karpicke & Roediger, 2008; Rickard & Pan, 2017; Roediger & Butler, 2011; Roediger & Karpicke, 2006a, 2006b; Rowland, 2014). The present account of the imaging findings is also in agreement with cognitive accounts that stress semantic/gist components (Carpenter, 2011) as well as memory strength (Rickard & Pan, 2017) as key factors underlying the TE.

From an educational perspective, the results in the current study further support the notion that retrieval practice relative study produces superior retention, and add information about the underlying mechanisms involved. As highlighted in the introduction, hippocampus is well known as a brain region important for learning and memory retrieval success per se. Here we found that despite the same behavioral outcome (correct answer), the degree of hippocampal engagement during retrieval success is *also* related to the “quality” of prior learning activity (study/retrieval practice). Moreover, as evident from the parametric modulation analysis, the number of successful retrievals during learning is a critical factor underlying the TE. Related to educational purposes, this emphasizes the importance of successive relearning which will tax brain regions such as hippocampus that are known to be important for memory formation and retention.

## 5 CONCLUSION

Our findings provide novel insights related to the TE by demonstrating that processing in both the anterior and posterior HC contributes to durable learning after retrieval practice. These findings are of educational significance as they contribute to a better understanding of *how* retrieval practice, relative study, improves memory retention of to‐be‐learned materials.

## CONFLICT OF INTEREST

The authors declare no conflict of interest.

## AUTHOR CONTRIBUTION

C.W‐H, B.J, and L.N designed research; C.W‐H performed research; C.W‐H, M.A, S.S, and L.N. analyzed data; and C.W‐H, M.A, S.S, B.J, and L.N wrote the paper.

### Peer Review

The peer review history for this article is available at https://publons.com/publon/10.1002/brb3.1909.

## Supporting information

Figure S1–S4‐Table S1

## Data Availability

The data that support the findings of this study can be available from the corresponding author upon reasonable request.
