# Supplementary material for: Retrieval practice facilitates learning by strengthening processing in both the anterior and posterior hippocampus
Source: Brain Behav. 2020 Oct 22;11(1):e01909. doi: 10.1002/brb3.1909 (PMC7821628; doi:10.1002/brb3.1909)
Supplement: Supplementary file 1 — Figure S1–S4‐Table S1 [file BRB3-11-e01909-s001.docx]

**This PDF file includes:**

- **Fig S1** illustrating the overlap between the main model in the ms and the control model
- **Fig S2** illustrating the overlap in HC activity when controlling for individual differences in performance
- **Fig S3** illustrating brain activation related to the parametric modulation analysis in the whole brain
- **Fig S4** illustrating the parametric modulation effect in the bilateral peaks in pHC
- **Table S1** is an activation table for the whole-brain contrasts presented

**
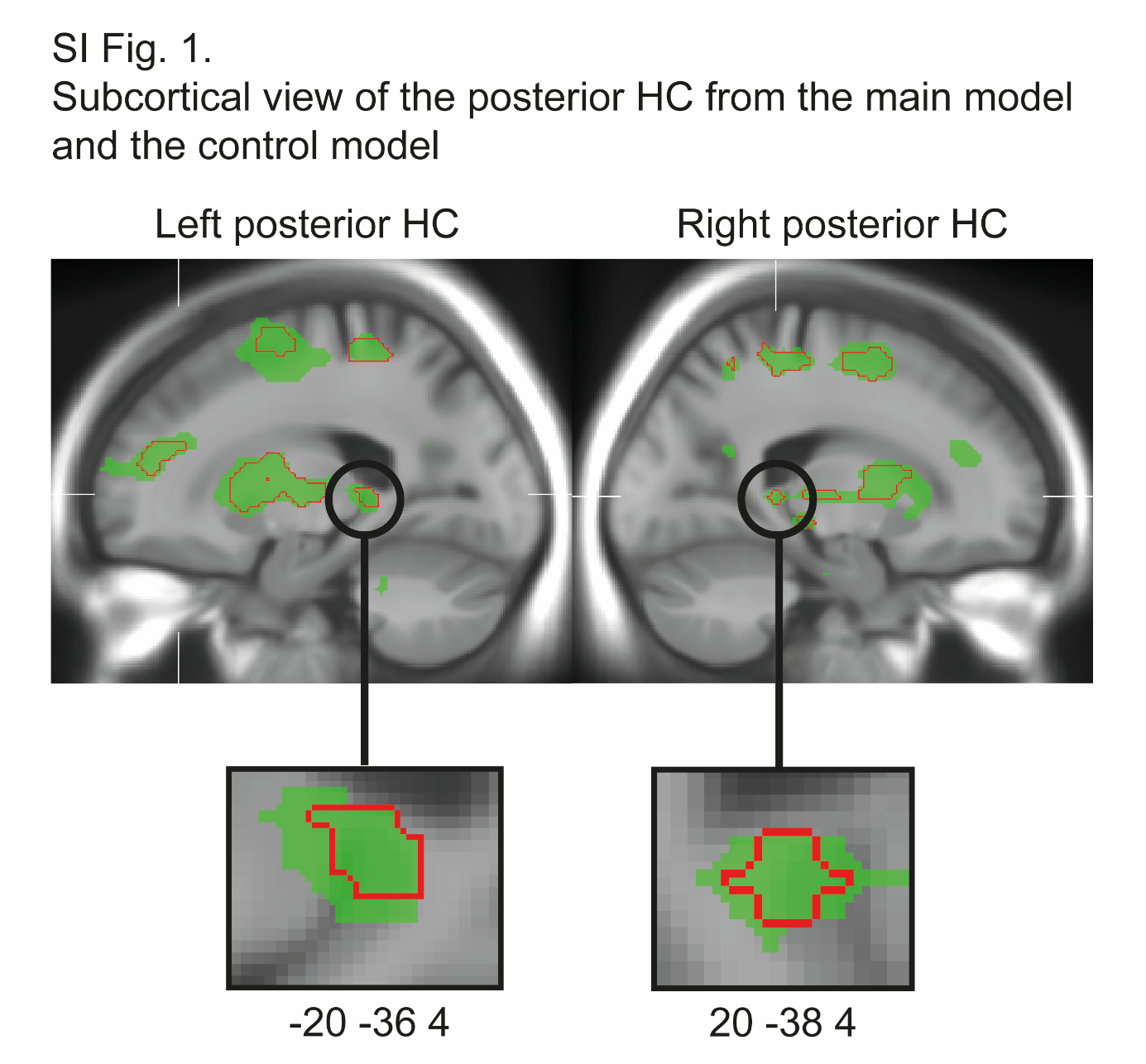
Supplementary Fig 1.** Overlap between the main model (red contour, *p* < .001) and the control model (green patch, *p* < .05 FDR) in the left and right posterior HC, respectively.


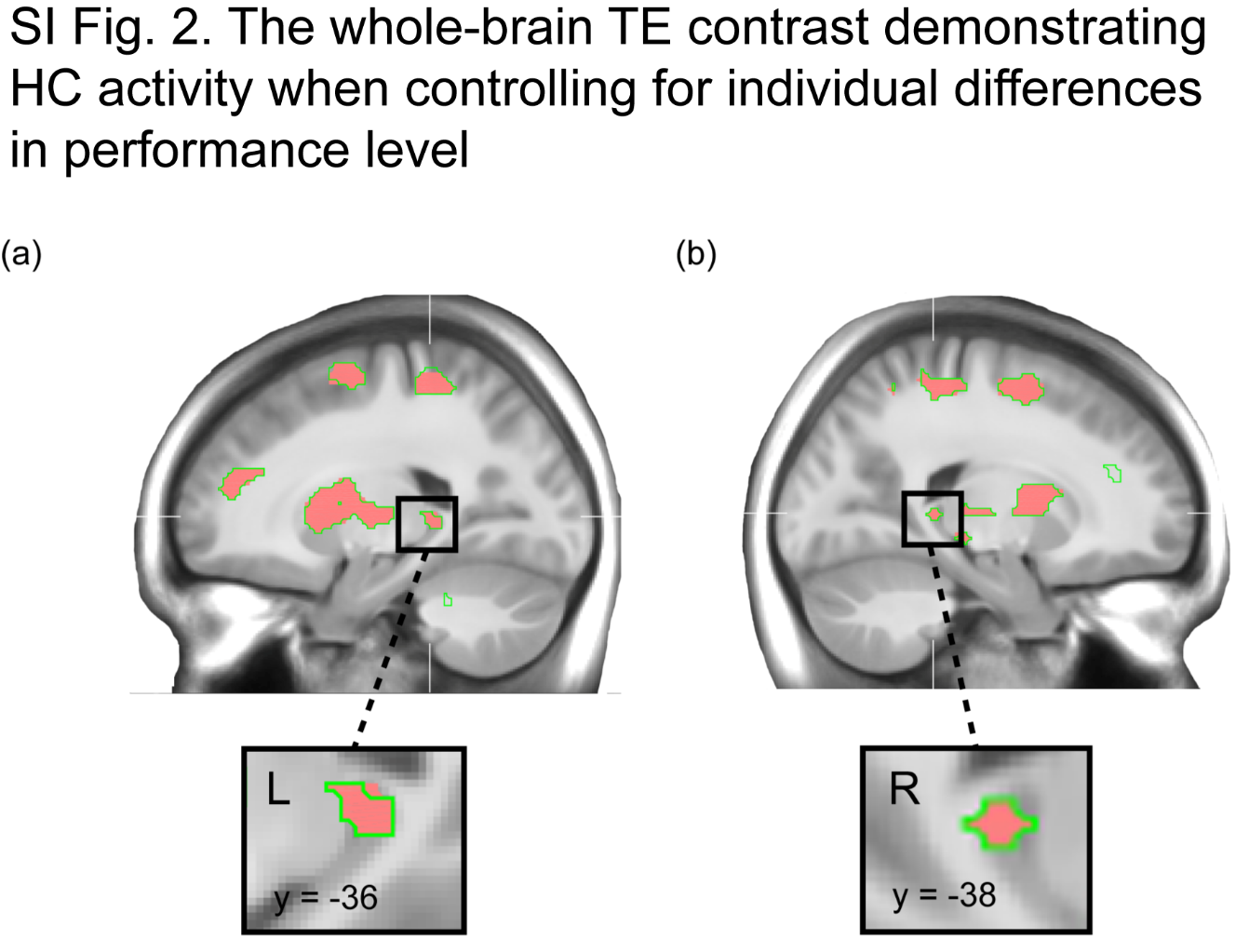


**Supplementary Fig. 2a-b.** The overlap in HC (remembered retrieval practice > remembered study; red patch) and when including individual differences in the magnitude of the TE (proportion correct T – proportion correct S) as a covariate of no interest in the same analysis (green contour). The zoomed images display overlap in (*a*) the left [-20 -36 4] and (*b*) right [20 -38 4] posterior HC, respectively (p < .001).

**
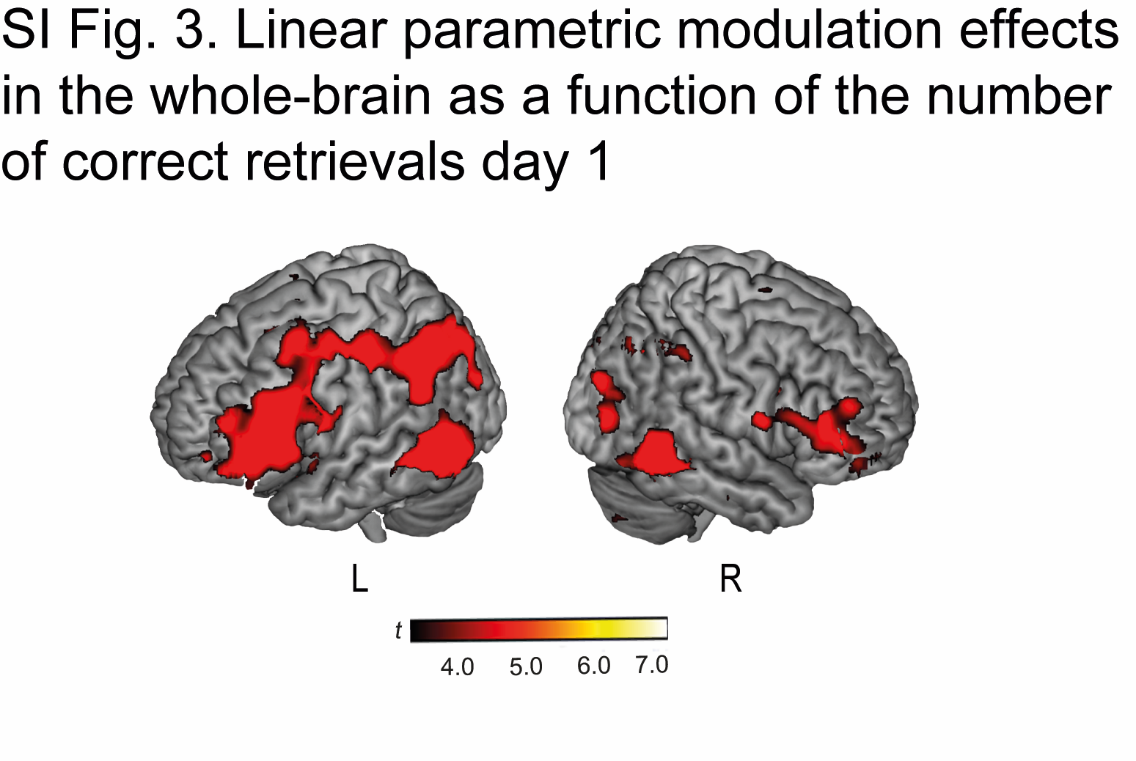
**

**Supplementary Fig. 3.** Linear parametric modulation effects in the whole-brain (*p* < .01 FDR corrected at the voxel level and *p* < .05 FWE at the cluster level).


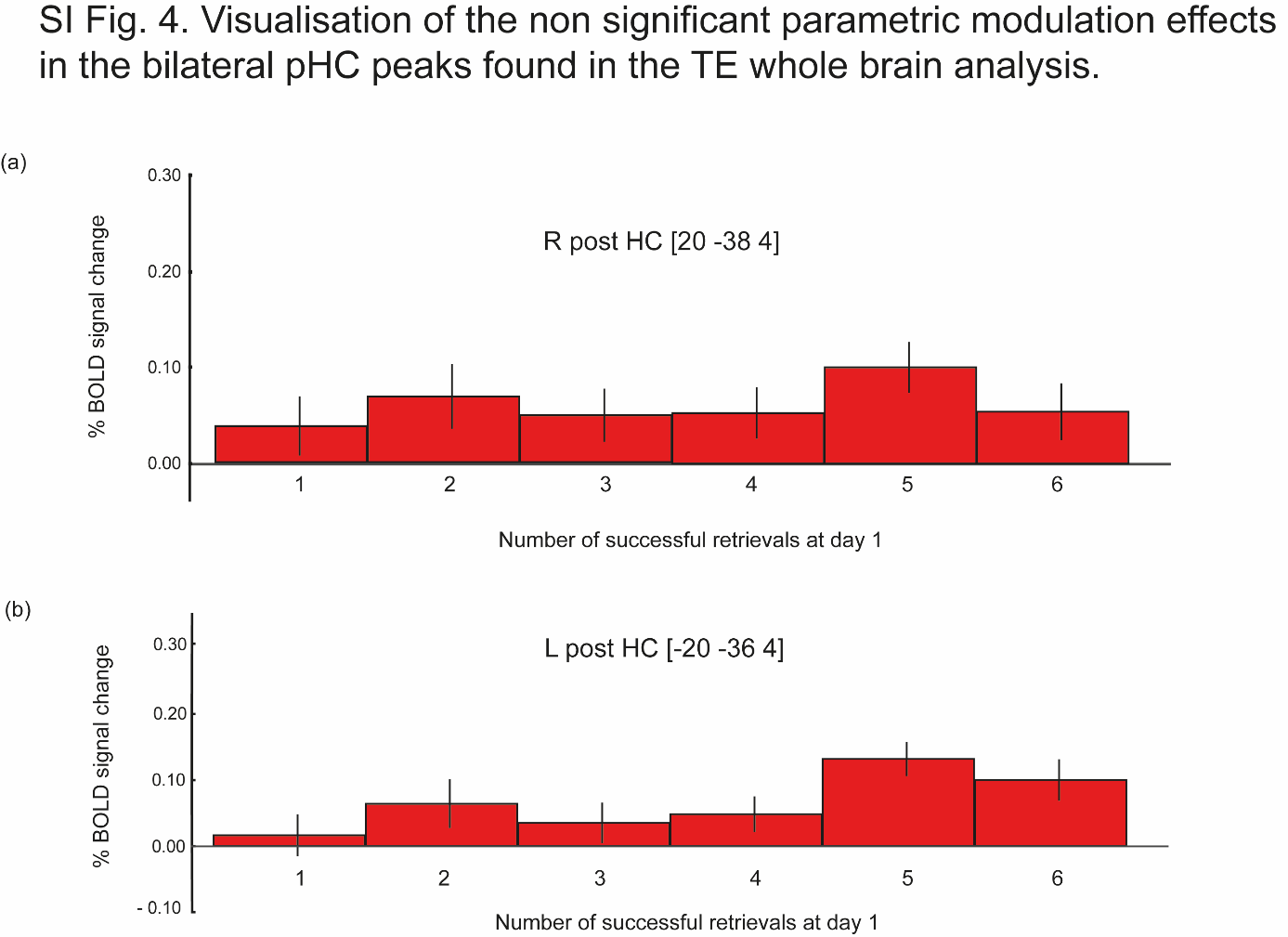


**Supplementary Fig. 4.** For visualization purposes we also plotted the outcome from the whole brain parametric modulation analysis in the (a) right pHC and (b) left pHC peaks found in the TE contrast.

**Supplementary Table 1.** Peak activation in significant clusters for the whole brain TE contrast (Remembered Retrieval practice > Remembered Study) and the whole brain parametric modulation analysis outside the hippocampal ROIs.

| Cluster | Local maxima | Hem |  | *x* | *y* | *z* | *Z*-score | Voxels (*k*) |
| --- | --- | --- | --- | --- | --- | --- | --- | --- |
|  |  |  |  |  |  |  |  |  |
| Remembered Retrieval practice > Remembered Study | | | | | | | | |
|  |  |  |  |  |  |  |  |  |
| 1 | Superior temporal gyrus | L |  | -60 | -42 | 20 | 3.55 | 8905 |
|  | Superior temporal gyrus | L |  | -62 | -50 | 18 | 3.45 |  |
|  | Precentral gyrus | L |  | -28 | -6 | 60 | 3.78 |  |
|  | Medial Precentral gyrus | L |  | -2 | -24 | 52 | 3.56 |  |
|  | Postcentral gyrus | L |  | -44 | -20 | 52 | 3.94 |  |
|  | Superior frontal gyrus | L |  | -18 | 0 | 66 | 3.91 |  |
|  | Supplementary motor area | L |  | -4 | -10 | 54 | 4.43 |  |
|  | Supplementary motor area | L |  | -6 | 6 | 60 | 3.41 |  |
|  | Precuneus | L |  | -12 | -44 | 56 | 4.04 |  |
|  | Middle frontal gyrus | R |  | 36 | 0 | 54 | 3.97 |  |
|  |  |  |  |  |  |  |  |  |
| 2 | Middle frontal gyrus | L |  | -26 | 46 | 20 | 4.49 | 1132 |
|  | Middle frontal gyrus | L |  | -36 | 34 | 32 | 3.95 |  |
|  | Middle frontal gyrus | L |  | -34 | 54 | 18 | 3.84 |  |
|  | Middle frontal gyrus | L |  | -42 | 42 | 18 | 3.40 |  |
|  |  |  |  |  |  |  |  |  |
|  |  |  |  |  |  |  |  |  |
|  |  |  |  |  |  |  |  |  |
| Parametric modulation: Number of successful retrievals during learning | | | | | | | | |
|  | | | | | | | | |
| 1 | Inferior parietal | L |  | -54 | -46 | 44 | 5.77 | 32.573 |
|  | Postcentral gyrus | L |  | -44 | -20 | 44 | 5.47 |  |
|  | Middle temporal | L |  | -60 | -42 | -12 | 5.26 |  |
|  | Middle temporal | L |  | -64 | -48 | 4 | 5.04 |  |
|  | Inferior temporal | L |  | -56 | -52 | -10 | 5.03 |  |
| 2 | Middle temporal | R |  | 66 | -48 | 2 | 4.87 | 1893 |
|  | Middle temporal | R |  | 56 | -42 | 2 | 4.52 |  |
|  | Inferior temporal | R |  | 66 | -42 | -10 | 4.41 |  |
|  | Superior temporal | R |  | 52 | -12 | -8 | 3.57 |  |
|  | Inferior temporal | R |  | 50 | -60 | -4 | 3.50 |  |
|  |  |  |  |  |  |  |  |  |
| 3 | Middle temporal | R |  | 50 | -76 | 12 | 4.19 | 615 |
|  | Middle occipital | R |  | 44 | -80 | 28 | 4.11 |  |
|  |  |  |  |  |  |  |  |  |
| 4 | Precentral | R |  | 60 | 6 | 18 | 4.06 | 1737 |
|  | Inferior frontal opercularis | R |  | 36 | 6 | 34 | 4.05 |  |
|  | Inferior frontal triangularis | R |  | 48 | 34 | 14 | 4.03 |  |
|  | Middle frontal | R |  | 46 | 50 | 24 | 4.03 |  |
|  | Inferior frontal triangularis | R |  | 54 | 44 | 8 | 3.92 |  |
|  |  |  |  |  |  |  |  |  |

*Note*. Up to 10 selected local maxima are reported for each cluster. Hem = Hemisphere. Coordinates (*x, y, z*) in MNI space (SPM12). *Z*-values at the peak voxel. One sample *t*-test: Voxel, *p* < 0.001, uncorrected. Cluster, *p* < 0.05 FWE corrected. Parametric modulation analysis: Voxel: *p* < 0.01 (FDR). Cluster: *p* < 0.05 FWE corrected.
